# Syntactic and Prosodic Phrasal Alignment in Naturalistic Language

**DOI:** 10.1111/cogs.70224

**Published:** 2026-05-17

**Authors:** Julie Bannon, Barbora Hlachova, Fernanda Ferreira

**Affiliations:** ^1^ Department of Psychology University of California Davis

**Keywords:** Prosody, Syntax, Prosodic phrasing, Language production, Speech planning

## Abstract

Prosody is an intrinsic element of language production, linking together multiple levels of linguistic representation to shape both the structure and interpretation of utterances. However, common theories of prosodic phrasing in spoken language often fail to capture factors associated with planning and recovery, as well as performance‐based effects related to working memory. Much of what we know about prosody, whether it be the features speakers are thought to generate or the ones listeners are believed to process, is based on forms that are atypical in spoken language. Recent developments in data analysis methods, however, allow for the efficient study of unrehearsed spoken language. The current work aims to develop more ecologically valid theories of prosody and its relationship to syntactic structure through the analysis of unrehearsed scene descriptions. Data from unrehearsed speech collected across four different studies showed only a weak to moderate relationship between prosodic phrasing and syntactic structure, such that the likelihood of a prosodic phrase boundary occurring at the end of a syntactic phrase was only slightly above chance. Additionally, correlations between occurrences of prosodic phrase boundaries and speech rate revealed that individuals who speak more slowly are likely to insert more prosodic phrase boundaries, indicating a relationship between prosodic phrasing and speech planning. The findings challenge some categorical approaches to prosody and suggest that prosodic phrasing may be a consequence of planning and recovery in language production, rather than a complement to syntactic phrasing. These results have implications for theories of language production and comprehension, formal theories of phonological structure, and computational tools for generating and interpreting language.

## 1 Introduction

### 1.1 What is prosody

#### 1.1.1 Definition and prosodic features

Prosody is a fundamental component of spoken language, shaping how utterances are structured and interpreted. It encompasses variations in rhythm, pitch, and speech rate that convey information beyond the lexical and syntactic content of an utterance. Prosody directly influences the timing, amplitude, and pitch contours of speech, making it an intrinsic feature of language rather than an optional embellishment (Cutler, Dahan, & Van Donselaar, 1997). For example, words that occur at the end of a syntactic phrase are typically lengthened and often followed by a pause, providing listeners with cues about the boundaries of syntactic and semantic units.

Researchers typically analyze prosody by examining rhythmic features such as word duration, stress patterns, and pausing. Some syllables are inherently longer or more prominent than others depending on their position within a sentence, and phrase‐final lengthening serves as an important cue to the locations of phrase boundaries. For instance, F. Ferreira (1993) demonstrated that phrase‐final words are consistently longer than words in phrase‐medial positions, suggesting that speakers maintain a rhythm that aligns with a metrical grid (Selkirk, 1984). Pitch variation is another critical aspect of prosody, as it helps distinguish statements from questions, expresses emotional or pragmatic intent, and also signals phrase boundaries. For example, English, as well as other languages, uses falling pitch contours to mark the end of a phrase and rising pitch to indicate continuation or uncertainty. These prosodic features, when combined with variations in duration and pauses, are used to segment the speech stream into prosodic phrases (i.e., groupings of words determined by prosodic features) that provide listeners with essential cues for parsing and interpreting speech.

Prosody is important for several reasons. First, it is a mandatory feature of spoken language. Even attempts to produce entirely monotone speech still result in subtle variations in pitch, duration, and intensity, underscoring its inextricable role in human communication and articulation. Second, prosody significantly enhances speech intelligibility. When prosodic cues are absent or atypical, as in robotic or early computer‐generated speech, the result is often speech that is unnatural and difficult to process. This suggests that prosody plays a critical role in spoken language comprehension, influencing how listeners parse and interpret sentences in real time.

In addition to its role in promoting speech clarity, prosody serves as a bridge between multiple levels of linguistic representation. While written language relies on punctuation and text formatting to clarify structure, spoken language depends on acoustic cues to guide comprehension. It encodes not only syntactic structure but also pragmatic and semantic information, and it reflects cognitive and physiological constraints on speech production. Unlike syntactic structures, which remain fixed regardless of delivery, prosodic phrasing can shift depending on speech rate, emphasis, or even physical effort. For example, a speaker who is out of breath may naturally produce shorter prosodic phrases, altering the rhythm and prosodic constituency of their speech, but the syntactic category of their utterance will remain unchanged. Finally, studying prosody provides valuable insights into the interplay between linguistic structure and cognitive constraints. While a noun phrase remains a noun phrase regardless of how it is spoken, prosodic phrasing is far more variable, often reflecting the speaker's need to manage planning, memory, and articulation. Understanding prosody, therefore, is not just relevant for linguistic theory; it is central to understanding the real‐time processes that shape spoken language, with implications for processing theories and real‐world technologies such as speech recognition and conversational agents.

Theories of prosodic phrasing generally fall into two categories: direct theories, which posit that prosodic features directly reflect syntactic structure, and indirect theories, which posit an intermediate level of prosodic constituency derived from, but not identical to, syntax (Bennett & Elfner, 2019). In direct theories, prosodic features such as phrase‐final lengthening, pausing, and pitch movements are seen as direct consequences of syntactic structure. This means that the placement of prosodic boundaries should align closely with syntactic boundaries, reflecting the underlying grammar of the sentence. Although direct theories are often dispreferred in favor of indirect theories in the present literature, early experimental work seems to support this view. For instance, studies have shown that words at the ends of phrases tend to be lengthened and followed by a pause (Cooper & Paccia‐Cooper, 1980). Conversely, flapping—a phonological process where /t/ and /d/ sounds become a soft “tap” in English—occurs more frequently across words when those words are not separated by a syntactic boundary.

Indirect theories, by contrast, assume that prosodic constituency is different from syntactic structure. One important difference is that indirect theories posit distinctly prosodic constituents such as phonological phrases (PPhs) and intonational phrases (IPhs), which are not syntactic phrases (F. Ferreira, 1993; Selkirk, 1986). Instead, IPhs and PPhs reflect a hierarchical prosodic structure, whereby IPhs represent larger prosodic groupings made up of smaller PPhs. According to indirect theories, the sentence *The old musician played the violin in the crowded cafe* might be structured as follows, where IPh indicates the end of an intonational phrase, comprised of smaller PPhs:

((The old musician)PPh (played)IPh (the violin)PPh (in the crowded café)PPh)IPh

The critical feature of this prosodic structure is that the main verb *played* groups with the subject of the sentence *the old musician* and not with its object. As a result, a prosodic boundary would occur after that sentence's main verb. In contrast, the sentence's syntactic structure groups the verb with its object, preventing a major prosodic boundary after the verb. Experiments designed to distinguish between the direct and indirect models demonstrated that variations in syntactic structure alone do not affect features such as word duration, contrary to the predictions of the direct model (F. Ferreira, 1993). Moreover, manipulations designed to induce a particular prosodic structure (i.e., contrastive prominence on a critical word) triggered a prosodic boundary, with measurable acoustic effects (F. Ferreira, 1993), which supports the indirect model. Work by Dannenberg, Werner, and Vainio (2016) further indicates that syntactic and prosodic phrasing in spontaneous spoken language follow different hierarchical structures, suggesting that prosodic structure is at least somewhat distinct from syntactic structure.

It is important to note that, even on indirect theories, some syntactic information is conveyed via prosody. For example, because PPhs typically end at the right edge of a syntactic phrase, prosodic features still mark certain syntactic boundaries. It is in part because of this marking of syntactic information that prosody has played a major role in psycholinguistic discussions of language comprehension, especially around questions of parsing. If prosody marks syntactic boundaries (either directly or indirectly), then a listener does not have to rely solely on abstract, silent syntactic knowledge to parse a sentence into syntactic constituents; instead, the listener can identify the boundary using the prosody of the sentence, potentially facilitating the task of parsing and comprehension.

A remaining consideration for theories of prosodic phrasing concerns how the strength of prosodic boundaries varies as a function of syntactic complexity. While the relationship is often assumed to be linear, with strong prosodic features associated with syntactic boundaries (e.g., Cooper & Paccia‐Cooper, 1980), research by Kentner, Franz, Knoop, and Menninghaus (2023) showed that phrase‐final lengthening of syllables varies depending on the strength of the linguistic boundary, where phrase‐final lengthening actually *decreased* at stronger boundaries. Kentner et al. identified five degrees of predicted boundary strength based on the placement of punctuation in a large speech corpus. They observed that final syllables were lengthened for words at a Level 2 boundary (which represents a small comma phrase in their coding scheme) but then decreased for words at Level 5 boundaries (which represent the end of a sentence in their coding scheme). This finding indicates that the prosody–syntax relationship is not strictly linear but rather that it changes over time as the utterance unfolds, a result our own findings reinforce (see Results section). One possible interpretation of this result is that prosodic phrasing is influenced by constraints on speech planning in addition to the structure of utterances (Wagner & Watson, 2010). Evidence suggests that planning scope typically encompasses individual phrases, and phrasal‐level planning may occur at the syntactic level (Allum & Wheeldon, 2007; Martin, Yan, & Schnur, 2014). The prosody–syntax relationship may therefore be impacted by the overall complexity of each utterance, reflected by the number of planning units needed to produce a complete sentence. Thus, theories of prosody–syntax alignment must consider how the relative strength of syntactic boundaries and the scope of planning affect the likelihood of a strong prosodic boundary rather than assuming an all‐or‐nothing approach in which a syntactic boundary automatically triggers a prosodic boundary.

#### 1.1.2 Performance units and constraints

While several theories suggest that prosodic phrasing is driven by linguistic factors such as syntactic structure, it is also influenced by factors that are not encompassed by linguistic theory in the strictest sense but rather relate to other physical or cognitive constraints on language production. For example, prosody also reflects the need for speakers to both recover from what was just said and plan for what is still to come (Wagner & Watson, 2010). This idea that prosody can be used to facilitate planning can be related to the need to manage working memory. Working memory is a limited capacity resource (e.g., Cowan, 2001; Cowan, 2010; Logie, 2011), which means that its contents must be continually updated. The limits of working memory are especially relevant to language processing because the capacity of working memory is often defined with respect to the number of phonological units (Baddeley, 2003). For example, it has been found that pauses tend to be longer before particularly complex phrases (F. Ferreira, 1991), suggesting that phrasing is affected by the need to update working memory. Although the relationship between prosodic phrasing and working memory has yet to be directly tested in the absence of linguistic constraints, Cowan and Elliot (2023) reported longer pauses following the third and sixth items during typed recall of nine‐item single‐digit sequences, providing evidence that spontaneous chunking in short‐term memory impacts the temporal structure of the output. While this finding pertains to typing rather than speech, it does indicate that people generate consistently sized chunks during recall from working memory. Other work shows that the length of prosodic phrases may be similar across a large and varied set of languages (Inbar, Grossman, & Landau, 2025), suggesting that prosodic phrasing may be primarily driven by the time‐course of language planning rather than the specific features of an individual language's grammatical forms.

Additionally, physical limitations, such as the need to breathe, can shape speech planning processes. Interestingly, evidence suggests that pauses to breathe in speech often occur at the end of a syntactic phrase rather than in the middle of syntactic units (Fuchs, Petrone, Krivokapić, & Hoole, 2013; Winkworth, Davis, Ellis, & Adams, 1994), and sounds in the speech stream associated with breathing can facilitate the identification of pauses during language comprehension (MacIntyre & Scott, 2022). This may guide listeners’ expectations about the rhythm of spoken language and suggests that the alignment between prosodic and syntactic phrasing may be guided by non‐linguistic constraints, including those related to respiration. These physical and cognitive constraints on language production likely play a role in prosodic phrasing. While syntactic units may sometimes function as planning units in speech, resulting in the alignment of prosodic cues and syntactic structure, the need to insert prosodic phrase breaks independently of syntactic boundaries can potentially lead to some misalignment between prosody and syntax.

### 1.2 Prosody in psycholinguistics

#### 1.2.1 Evidence from the laboratory

The linguistic theories supporting the idea that prosodic and syntactic phrases align in spoken language are widely reflected in psycholinguistic literature, where researchers commonly assume listeners can infer syntactic structure from prosodic cues. This assumption is backed by considerable evidence indicating that listeners use prosodic cues to interpret relative clauses (e.g., Lee & Watson, 2011; Pynte, 1996), to resolve other types of syntactic ambiguity (e.g., Engelhardt, Ferreira, & Patsenko, 2010; Snedeker & Trueswell, 2003), and to estimate the end of conversational turns (e.g., Bogels & Torreira, 2015; Bogels & Torreira, 2021). Further, sentence processing is facilitated when prosodic cues align with syntactic structure, such as through increased word durations or increased pauses at the end of a syntactic phrase (e.g., Engelhardt et al., 2010; Kjelgaard & Speer, 1999), leading to more successful sentence parsing when syntactic phrase boundaries are prosodically marked (Degano, Donhauser, Gwilliams, Merlo, & Golestani, 2024). The alignment of prosodic phrase boundaries with syntactic structure therefore represents a fundamental relationship in spoken language that supports listeners’ ability to successfully interpret linguistic input. However, in studies of language production, the use of prosodic cues to disambiguate syntactic structure often depends on speaker's awareness of the syntactic ambiguity, suggesting that prosody may be inconsistently used by speakers to aid listeners (e.g., Gao & Gu, 2024; Kraljic & Brennan, 2005; Snedeker & Trueswell, 2003).

The specific prosodic cues that facilitate sentence parsing are central to the debate regarding the prosody–syntax interface. While some studies focus on individual elements of prosody such as pitch (e.g., Lee & Watson, 2011) or duration (e.g., Shattuck‐Hufnagel & Turk, 1998), many focus on some combination of prosodic cues to estimate prosodic boundaries (e.g., Buxo‐Lugo & Watson, 2016; Degano et al., 2024; Kentner et al., 2023). In order to tease out the contributions of prosodic features in sentence parsing, traditional laboratory‐based experiments often experimentally manipulate prosodic cues to ensure that the acoustic signal is carefully controlled. For example, the insertion of pauses at the end of syntactic clauses, or adjustments to pitch contours, can be experimentally controlled to investigate specific cues that facilitate decoding of syntactic structure. A similar approach is reflected in the language production literature, which often employs experimental paradigms that invite speakers to read pre‐written items out loud, often allowing for multiple attempts to encourage smooth renditions. While these approaches have provided valuable evidence about the role of prosody in language production and comprehension, they also may reflect the type of speech generated under close to ideal speaking conditions. Theories of prosodic phrasing should also account for the inherent flexibility in everyday language production that allows speakers to quickly plan and generate their utterances in response to a wide range of communicative situations, and should attempt to explain the errors and corrections that characterize unrehearsed speech.

#### 1.2.2 Current measures of prosody and syntax

The reliance on idealized stimuli can be attributed to limitations in psycholinguistic approaches to handling speech production data, which have historically relied on hand‐coding by trained research assistants. For example, the Tones and Breaks Indices (ToBI) system represents an early attempt to systematically identify prosodic boundaries using human annotators (Pitrelli, Beckman, & Hirschberg, 1994; Silverman et al., 1992). This system involves the labeling of prosodic events across tones within words, as well as breaks between words, to identify the prosodic structure of utterances. A similar system, the Rhythm and Pitch (RaP) system, was developed in the mid‐2000s to account for rhythmic features of speech by including annotation of syllables in addition to tones (Breen, Dilley, Kraemer, & Gibson, 2012; Dilley & Brown, 2005). Such annotation systems were used widely throughout the 1990s and 2000s to identify the prosodic structure of spoken language (e.g., Cho & Keating, 2001; Dilley, Shattuck‐Hufnagel, & Ostendorf, 1996; Snedeker & Trueswell, 2003). While prosodic annotation systems proved useful for examining prosody in more naturalistic data from speech corpora, they require considerable time and effort to effectively annotate speech samples, and remain limited by the use of human annotators who often rely on subjective interpretations and simple heuristics, resulting in poor reliability (Jun, 2022; Wightman, 2002).

More recently, researchers have made strides in identifying the prosodic boundary strength of individual words using unsupervised identification of prosodic features of speech (e.g., Biron et al., 2021; Dannenberg et al., 2016; Suni, Šimko, Aalto, & Vainio, 2017). One such example, the Wavelet Prosody Toolkit, developed by Suni et al. (2017), makes use of continuous wavelet transformation to extract the pitch, duration, and energy of individual words and uses those measures to calculate the prosodic prominence and boundary strength of individual words in the speech stream. Using unsupervised *k*‐means clustering to identify strong and weak prosodic boundaries, Suni et al. demonstrated that this toolkit accurately approximates prosodic boundary identification from trained human annotators using ToBI, while taking into account multiple prosodic features using unsupervised machine learning. This approach provides a powerful tool for the automatic detection and identification of prosodic boundaries and has been employed in recent research demonstrating universal rhythms in speech across 48 languages (Inbar et al., 2025). Importantly, the development of the Wavelet Prosody Toolkit enables researchers to accurately calculate prosodic boundary strengths and cluster words into separate distributions representing strong and weak boundaries, without introducing additional researcher bias.

In addition to advances in the identification of prosodic boundaries in speech, modern developments in natural language processing have enabled researchers to efficiently parse large speech corpora and identify the underlying syntactic structure of unscripted speech (e.g., Kitaev & Klein, 2018; Qi, Zhang, Zhang, Bolton, & Manning, 2020). Together, these tools provide objective measures of both the prosodic and syntactic structure of speech, which allow for more effective investigations of the prosody–syntax relationship. A recent study by Degano et al. (2024) used the natural language processing tool spaCy (Honnibal et al., 2020) along with the Berkeley Neural Parser (Kitaev & Klein, 2018) to identify syntactic closing phrase boundaries and the Wavelet Prosodic Toolkit (Suni et al., 2017) to extract prosodic boundary strength for each word in four different TED Talks. Although the main goal of their study was to use magnetoencephalography to examine whether prosodic cues facilitate decoding of syntactic boundaries during language comprehension, their methods made significant strides in characterizing the degree of alignment between syntactic and prosodic features of spoken language. Using a two‐gamma mixture model to cluster words into strong and weak prosodic boundaries, their results showed that 72% of words produced with a strong prosodic boundary were words that terminated a syntactic phrase. However, for words produced with a weak prosodic boundary, 44% occurred at the end of a syntactic phrase, suggesting that a large number of syntactic boundaries may go unmarked. Additionally, Degano et al. found that the alignment of prosodic boundaries with the edge of syntactic phrases facilitated decoding of syntactic structure, lending support to the validity of this method for accurately classifying prosodic boundaries in spoken language.

### 1.3 The current study

The results from the Degano et al. (2024) study showing only moderate alignment between syntactic and prosodic boundaries are somewhat unexpected given the standard assumption in linguistics and psycholinguistics that the right edges of syntactic phrases are obligatorily marked by cues associated with a prosodic boundary. However, the results must be treated as only suggestive, since the main purpose of the study was not to investigate syntax‐prosody alignment but rather to examine neural encoding of syntactic phrases based on the presence or absence of prosodic cues. In addition, although the use of TED Talks is an important methodological innovation since the language produced was not generated for the specific purpose of testing psycholinguistic theories, the speech samples have some important limitations: TED talks are typically highly rehearsed, professionally delivered, and often memorized. In contrast, everyday speech is typically produced in situations that allow for less extensive planning and rehearsal.

The primary goal of the current work is to investigate the extent to which prosodic and syntactic boundaries align in unrehearsed spoken language. As discussed, current theories regarding the prosody–syntax interface often posit that prosodic phrases map onto syntactic structure in such a way that the end of a prosodic phrase aligns with a closing syntactic phrase boundary, thus facilitating sentence parsing in language comprehension. To date, evidence for the prosody–syntax relationship in spontaneous, unscripted speech comes primarily from studies that rely on identification of prosodic and syntactic boundaries (e.g., Dannenberg et al., 2016; Degano et al., 2024; Heldner & Megyesi, 2003) but do not incorporate continuous measures of boundary strength. The results of Kentner et al. (2023), however, revealed that the relationship between prosodic and syntactic boundaries is not strictly linear but rather varies over the course of the utterance. In the current work, we therefore consider not only the alignment of prosodic and syntactic boundaries but also how the relationship between boundary strengths might affect the likelihood of a syntactic boundary receiving prosodic emphasis.

We re‐analyzed results from four separate datasets of unscripted, verbal descriptions of complex visual scenes. These datasets were collected as part of a separate line of research investigating the allocation of visual attention to guide the production of scene descriptions, and thus constitute unrehearsed and spontaneously generated spoken language. The availability of these datasets further allowed us to examine the prosody–syntax interface across different populations (three datasets with younger adults and one with older adults) and variations in task instructions (i.e., “provide a description of the scene” vs. “describe what the average person might do in this scene”). In each study, participants were given 30 s to produce their descriptions of 30 unique visual scenes. The descriptions produced across these four datasets resulted in 1800 min, or 30 h, of unscripted, unrehearsed spoken language.^1^

## 2 Method

### 2.1 Participants

Participants were 91 college‐aged adults^2^ recruited from the undergraduate community at the University of California, Davis, who participated for course credit, and 30 older adult volunteers (*age range* = 66–82, *mean age* = 72.63, *SD* = 4.94) recruited from the broader Davis community. All participants reported learning English before the age of 5, had normal or corrected to normal vision, and no known color‐blindness. Additionally, the older adults reported no known history of dementia. All participants provided written consent to participate in the research.

### 2.2 Materials

**Fig. 1:**
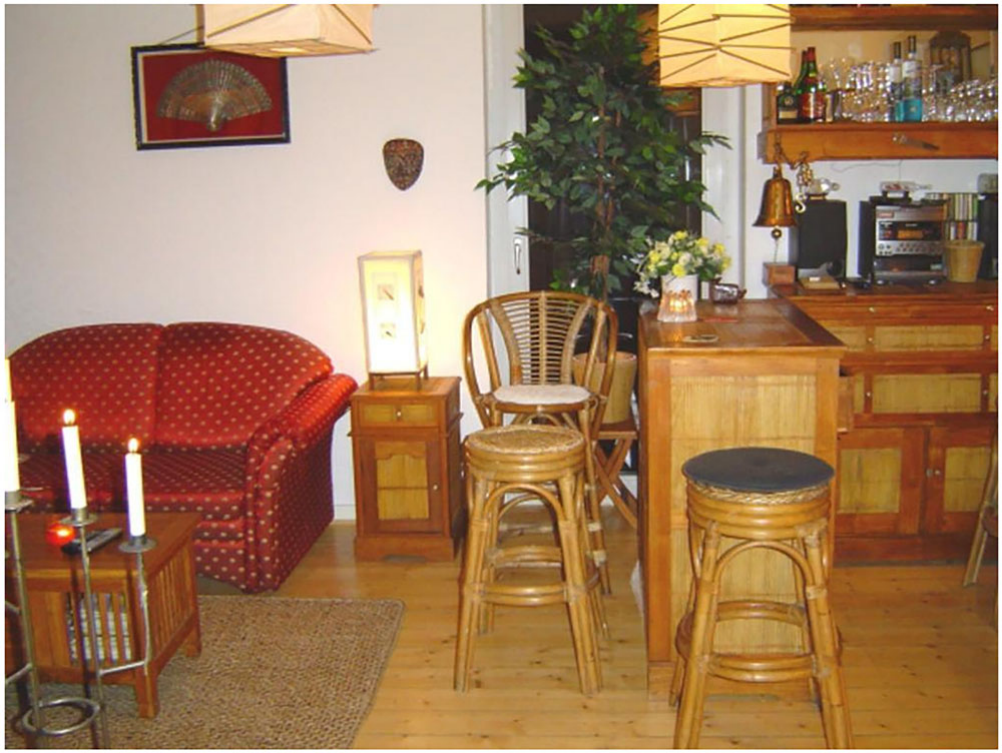
Example experimental display.

The stimuli consisted of 30 real‐world visual scenes developed by Henderson, Hayes, Rehrig, and Ferreira (2018). The scenes consisted of both indoor (*N* = 20) and outdoor (*N* = 10) environments (see Fig. 1 for an example). The indoor scenes depicted various room types (e.g., kitchens, living rooms, desk areas), and the outdoor scenes depicted different building types (e.g., houses, businesses) or street views. None of the scenes contained people. Each scene was displayed at 1024 × 768 pixels.

### 2.3 Procedure

Each dataset analyzed here was collected following the same general procedure but with small variations in the task instructions. Across all four studies, participants were brought into the lab to participate in an eye‐tracking experiment designed to examine the relationship between visual attention and linearization in language production. Following calibration of the eye‐tracking apparatus, participants were told they would see a series of scenes presented one at a time, and their task was to provide a verbal description of each scene. At the beginning of each trial, participants focused on a fixation dot in the center of the screen to check the eye‐tracker's calibration. Following this check, the scene was displayed for 30 s, in which the participants produced descriptions while visually examining the scenes. The scenes were presented to each participant in a unique pseudo‐random order to ensure that no two scenes from the same category were presented consecutively. The current investigation focuses on the verbal descriptions, and the eye‐tracking data are not considered in the present work. Further details of the procedure can be found in Henderson et al. (2018) and Rehrig, Hayes, Henderson, and Ferreira (2023). See Table 1 for a summary of the four experiments.

**Table 1:** Description of datasets analyzed in the current study

Age Group	Experiment Name	Citation	Task Instructions
Young adults	Scene Descriptions: YA	Rehrig et al. (2023)	Describe the visual scene.
	Time Pressure: YA	Unpublished data	Describe the visual scene. Begin your description before the deadline (1–2 s).
	Action Descriptions: YA	Henderson et al. (2018)	Describe what the average person would be inclined to do in the scene.
Older adults	Scene Descriptions: OA	Rehrig et al. (2023)	Describe the visual scene.

### 2.4 Data preparation

Scene descriptions were transcribed using automated transcription tools and manually corrected by a research assistant naive to the research hypotheses. The audio recordings were processed using the Montreal Forced Aligner (McAuliffe, Socolof, Mihuc, Wagner, & Sonderegger, 2017) to align the voice recordings with the transcriptions and then manually corrected by research assistants using Praat (Boersma & Weenink, 2024).

Prosodic and syntactic features of the speech stimuli were extracted following the methods described in Degano et al. (2024). First, the transcriptions were annotated using the natural language processing tool spaCy along with the Berkeley Neural Parser to determine the syntactic structure of the scene descriptions. The words were first tokenized and tagged for part of speech and then parsed to identify syntactic constituencies in the speech data. Certain compound words or contractions that were not represented as a single token were discarded from further analyses. This resulted in the removal of 3.5% of words across all four datasets.

The constituency parse identifies the number of syntactic closing nodes for each word in the scene descriptions, where syntactic closing nodes represent the number of syntactic phrases that end at that word. For example, the sentence *“The boy walked to school”* contains a noun phrase (i.e., “The boy”), a verb phrase (i.e., “walked to school”), and a prepositional phrase (i.e., “to school”), resulting in the following syntactic parse:

(S (NP (The) (boy)) (VP (walked) (PP (to) (school))))

In this parse, each word receives one syntactic closing node representing a word boundary (identified by a right closing bracket), and words that occur at the edge of syntactic phrases receive an additional number of brackets corresponding to the number of syntactic phrases that end at that word. The number of closing syntactic nodes was counted for each word, and words that had a node count greater than one (where one closing node represents a word boundary) were considered a syntactic boundary.

Next, the prosodic boundary strength was computed using the Wavelet Prosody Toolkit (https://github.com/asuni/wavelet_prosody_toolkit; Suni et al., 2017). The toolkit uses continuous wavelet transformation to calculate f0, energy, and duration of each word in a given audio file and corresponding Praat text grid, and then sums these values to provide a measure of prosodic boundary strength. All recordings across the experiments were normalized to ensure minor variations in speaker volume did not influence the prosodic analysis. Following the methods of Suni et al. (2017), the range of possible pitch values was adjusted depending on the gender of the speaker (males: 50–350 Hz; females: 100–400 Hz), and the computed values for f0, energy, and duration were weighted based on the default settings (f0: 1; energy: 1; duration: 0.5). Words with a computed boundary strength equal to 0 or with a total word duration that was less than 100 ms were removed from further analyses in order to discard words with insufficient acoustic variation.

**Fig. 2:**
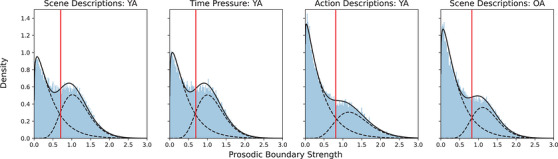
Distribution of prosodic boundary strength across all four datasets. The solid black line represents the probability density function, and the dashed lines reflect the two underlying distributions for strong and weak boundaries. The solid red line represents the cutoff value for strong prosodic boundaries.

Following the approach of Degano et al. (2024), density plots were created for the full range of prosodic boundary strengths in each experiment (see Fig. 2). The plots were created by selecting 1000 values from the full range of prosodic boundary strengths across each dataset and then calculating a probability density function to represent the likelihood of a given value of prosodic boundary strength. The data were then modeled using a two‐gamma mixture model to extract two underlying distributions representing values classified as strong and weak prosodic boundaries. Mixture models are typically used to model data that are assumed to be made up of two or more categories that cannot be directly observed, and enable the data to be clustered based on these underlying categories (Young, Chen, Hewage, & Nilo‐Poyanco, 2019). Finally, the likelihood of a boundary strength value belonging to each distribution was determined and a cutoff value was calculated based on identifying the lowest value that was predicted to belong to the distribution representing strong prosodic boundaries. This value was then predicted 500 times for each dataset, and the final cutoff value was determined by taking the average of all simulations.^3^ For the main analyses, the data were subset to include all remaining words that coincided with a syntactic boundary. A sample of words that did not coincide with a syntactic boundary was then randomly selected to match the number of words that did coincide with a boundary. This was done to ensure an equal number of words in each category in the analysis. The python code for the data preparation and all subsequent analyses is available through the Open Science Framework (https://osf.io/csz89/overview).

## 3 Results

### 3.1 Alignment of prosodic and syntactic boundaries

**Fig. 3:**
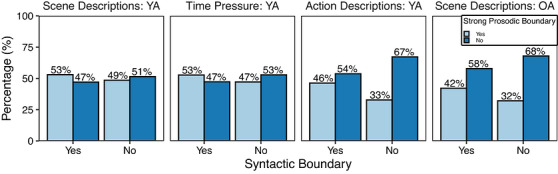
Alignment of syntactic and prosodic boundaries across all four datasets.

The first analysis aimed to examine the hypothesis that strong syntactic boundaries are consistently marked by a prosodic boundary. Because the theoretical predictions are unidirectional—a syntactic boundary should align with a prosodic boundary, but prosodic boundaries may not necessarily align with a syntactic boundary since they may be introduced for other reasons (e.g., to introduce contrastive prominence)—we focus this analysis on the proportion of words representing a syntactic boundary that aligned with a corresponding prosodic boundary. First, for each of the four individual datasets, the data were grouped into four categories to determine the alignment between syntactic and prosodic boundaries, as follows: syntactic boundary with a prosodic boundary; syntactic boundary with no prosodic boundary; no syntactic boundary with a prosodic boundary; and no syntactic boundary with no prosodic boundary. As can be seen in Fig. 3, across all four datasets, syntactic boundaries are aligned with a prosodic boundary only slightly more than 50% of the time in the Scene Description: YA and Time Pressure: YA datasets. Alignment of prosodic and syntactic boundaries was even lower in the Action Description: YA and Scene Description: OA datasets (46% and 42%, respectively).

We employed a one‐tailed binomial test to determine whether the likelihood that a syntactic boundary corresponded with a prosodic boundary was greater than chance (Fig. 3, the left pale blue bars representing a syntactic boundary). Cohen's *h* was then calculated to determine the effect size of the alignment. Because current theories are agnostic concerning whether a word with no syntactic boundary will be prosodically marked (as noted above), we analyze only the words that coincided with a syntactic boundary. In the two datasets with young adult scene descriptions (Scene Descriptions: YA and Time Pressure: YA), alignment of syntactic boundaries and prosodic boundaries was greater than chance (probability of alignment = 0.53, *p* <  .001, for both datasets), but effect sizes were quite small (0.05 and 0.06 for Time Pressure: YA and Scene Descriptions: YA, respectively). We do not report the results of the binomial test for the Action Descriptions: YA and Scene Descriptions: OA datasets as these both had alignment percentages below 50% and thus cannot occur at a rate greater than chance. Because our primary interest in this research was to investigate the relationship between prosodic phrasing and syntactic structure, we do not further consider how additional variables may improve the prosody–syntax alignment here. Additional analyses examining how the inclusion of part of speech and word position in the utterance influences the predicted alignment of prosodic and syntactic boundaries can be found in the Supporting Information.

### 3.2 Prosodic and syntactic boundary strength

The previous analysis used binary classifications of prosodic and syntactic boundaries to determine alignment. Given that both syntactic and prosodic boundary strength are continuous variables, a simple binarization of the alignment between these linguistic features may be insufficient to capture the complex relationship between prosody and syntax. While previous theories are typically agnostic to the influence of syntactic boundary strength on the alignment of prosodic and syntactic phrasing, evidence suggests that prosodic boundary strength increases with the increasing number of closing syntactic nodes but may then decrease as the number of nodes piles up (Kentner et al., 2023). If the relative prosodic boundary strength increases with syntactic boundary strength, this may suggest that words occurring at the end of weak syntactic boundaries do not receive enough prosodic emphasis to result in a strong prosodic boundary. To investigate this, we next examined whether prosodic boundary strength increases as a function of the number of closing nodes at syntactic boundaries.

We began by constructing linear mixed effects models with prosodic boundary strength as the dependent variable and closing node count as a continuous predictor variable. The analyses were performed on a subset of the data where the number of closing nodes was 10 or less to ensure sufficient data in each category. Participant and Trial were included as random effects in all models. Three separate models were constructed for each dataset to test the relationship between syntactic and prosodic boundary strength to determine whether the relationship is best explained by a linear, quadratic, or cubic fit. All three models revealed that the number of closing nodes significantly predicted the strength of prosodic boundaries. However, a model comparison using the Chi‐Square goodness of fit test revealed that the relationship is best explained through a cubic fit of the data. Table 2 provides the chi‐square values for the model comparison.

**Table 2:** Model comparisons for linear, quadratic, and cubic fit of the relationship between syntactic and prosodic boundary strength

Dataset	*Χ^2^*	*df*	*p*
Scene Descriptions: YA	69.97	1	<.001
Time Pressure: YA	84.58	1	<.001
Action Descriptions: YA	27.22	1	<.001
Scene Descriptions: OA	14.65	1	<.001

**Fig. 4:**
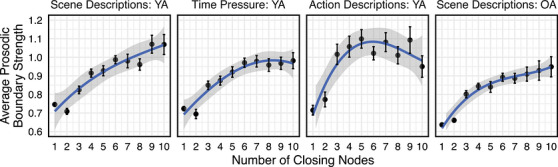
Cubic relationship between syntactic and prosodic boundary strength. Error bars represent the standard error of the mean.

Fig. 4 shows the cubic relationship between prosodic boundary strength and number of closing syntactic nodes. This pattern suggests that prosodic boundary strength increases with the number of syntactic closing nodes but levels off and begins to drop off at higher levels of syntactic boundary strength, consistent with Kentner et al. (2023). Further, the average prosodic boundary strength is relatively weak when only one closing node is present (i.e., a word boundary plus at least one closing syntactic node, denoted as two closing nodes in Fig. 4) and may indicate that weak syntactic boundaries are less likely to receive prosodic emphasis.

### 3.3 Alignment of prosodic and strong syntactic boundaries

**Fig. 5:**
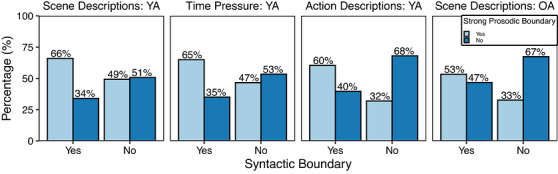
Alignment of syntactic boundaries with at least two closing syntactic nodes and prosodic boundaries.

Given the result that the average prosodic boundary strength is relatively weak in positions that receive a low number of syntactic closing nodes, we next examined whether establishing a higher cutoff of at least two closing syntactic nodes beyond a word boundary resulted in greater alignment between prosodic and syntactic boundaries. The data were again subset to include all remaining words that coincided with a “strong” syntactic boundary, and an equal number of words that did not coincide with a syntactic boundary were randomly selected to ensure an equal number of items in each category. As shown in Fig. 5, a higher threshold of syntactic boundary strength leads to better alignment of prosodic and syntactic boundaries. One‐tailed binomial tests confirmed that alignment was greater than chance for all datasets (*p*s < .001), but effect sizes remained small across all four datasets (Scene Descriptions: YA: *h* = 0.33; Time Pressure: YA: *h* = 0.30; Action Descriptions: YA: *h* = 0.21; Scene Descriptions: OA: *h* = 0.07).

### 3.4 Prosodic boundaries and speech rate

A final consideration concerns how prosodic phrasing is used beyond its relation to syntactic structure. As discussed in the introduction, prosody is used not only to cue syntactic structure but is also guided by cognitive constraints on speech planning. In this final analysis, we examine the relationship between the proportion of prosodic boundaries produced across the scene descriptions and the overall speech rate. A faster speech rate may reflect more efficient planning processes, compared with slower speech rates, where speakers may be planning in smaller semantic or syntactic chunks. A negative relationship between the proportion of prosodic boundaries and speech rate would therefore suggest that prosodic phrasing varies as a function of linguistic planning units.

**Fig. 6:**
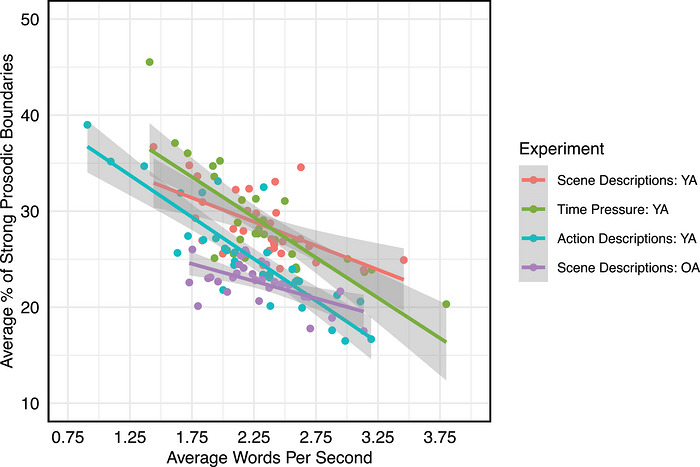
Relationship between average proportion of strong prosodic boundaries and average speech rate. Each data point represents a participant.

The average proportion of words identified as prosodic boundaries was calculated for each trial in the full datasets, and speech rate was calculated by determining the number of words per second for each trial. Across all experiments, there was a significant, negative correlation between average speech rate and average proportion of strong prosodic boundaries (see Fig. 6; see Table 3 for a summary of the results), suggesting that prosodic phrasing is influenced by cognitive constraints on language production, and in particular, the factors that allow a speaker to speak more quickly or require the speaker to take more time to retrieve and produce words.

**Table 3:** Summary of Pearson correlations between speech rate and proportion of strong prosodic boundaries

Dataset	*r*	*df*	*p*
Scene Descriptions: YA	−0.59	28	.001
Time Pressure: YA	−0.78	28	<.001
Action Descriptions: YA	−0.86	29	<.001
Scene Descriptions: OA	−0.58	28	.001

## 4 Discussion

This work investigated the relationship between prosodic phrasing and syntactic structure in unrehearsed spoken language. Following the methods developed by Degano et al. (2024), we identified prosodic and syntactic phrase boundaries in unscripted speech produced in response to four scene description tasks. Across all four datasets, we found prosodic and syntactic boundaries aligned slightly above chance only for young adult participants who produced descriptions of the visual scene. However, when asked to describe actions that could be taken within those same scenes, young adults showed less than 50% alignment of prosodic and syntactic boundaries. Further, older adults also showed less than 50% alignment when asked to describe the scenes. Raising the threshold to define syntactic boundaries (thus restricting the analyses to stronger syntactic boundaries) did increase alignment across all four datasets, but the effect sizes remained small, suggesting that alignment between prosodic and syntactic boundaries is relatively weak in unrehearsed spoken language. Additionally, analysis of the relationship between prosodic and syntactic boundary strength revealed that these linguistic features do not share a linear relationship, but rather that the average prosodic boundary strength increases with increasing syntactic boundary strength and then drops at the higher levels of syntactic boundary strength, as also reported by Kentner et al. (2023). Finally, correlations between speech rate and the average number of prosodic boundaries per description revealed that speakers produced fewer prosodic boundaries at higher speech rates—that is, in faster speech, more words were contained in each prosodic phrase—thus indicating that prosodic phrasing may be linked to aspects of speech planning beyond syntactic structure.

These findings stand in contrast to both direct and indirect theories of prosody. Unlike direct theories of prosody, which posit that prosodic features are a direct reflection of syntactic structure, the current results indicate that the degree to which prosodic phrasing reflects syntactic structure is only slightly above chance. More importantly, indirect theories, which argue that the hierarchical structure of prosodic phrasing is not dependent on the strength of syntactic phrase boundaries, are not supported by the findings that alignment of syntactic and prosodic boundaries increases when a higher threshold of syntactic boundary strength is considered. This low level of alignment between prosodic and syntactic boundaries carries implications for psycholinguistic research that shows listeners can use prosodic cues during language comprehension to aid in parsing syntactic structure. Critically, if prosodic cues do not consistently align with syntactic boundaries in everyday speech, then prosodic phrasing may not be a reliable cue to identify syntactic boundaries. Instead, the placement of prosodic boundaries may be a speaker‐oriented aspect of spoken language that reflects the system's need to plan and recover rather than serving as a source of useful cues to a speech partner. This would be consistent with research showing that the language production system favors the speaker (e.g., V. S. Ferreira & Dell, 2000; Piantadosi, Tily, & Gibson, 2012), at least in part due to the inherently more difficult task of producing speech in contrast to comprehending it (e.g., Levinson, 2000).

In line with the theory that prosodic phrasing reflects planning, the results show that the number of prosodic boundaries generated is inversely related to speech rate: When individuals speak more slowly, more words of an utterance are prosodically marked. Conversely, when speech is produced more quickly, a smaller proportion of words in the utterance represent prosodic boundaries, indicating that more words are contained in each prosodic phrase. This may reflect temporal constraints on speech planning, where prosodic boundaries are produced in units of similar absolute duration, and slower speech results in fewer words produced in each prosodic or temporal chunk. Interestingly, Inbar et al. (2025) demonstrated that across 48 languages, prosodic phrases typically begin every 1.6 s, suggesting that the temporal structure of speech may be universal across languages and speakers. Such temporal constraints on speech planning can influence the extent of alignment between prosodic and syntactic phrasing and suggest that prosodic phrasing is driven by domain‐general cognitive processes. Specifically, if prosodic phrasing is driven at least in part by speech planning, then the relation between prosody and syntactic structure may be a consequence of planning scope—which typically encompasses individual phrases (Allum & Wheeldon, 2007; Martin et al., 2014)—rather than a direct relationship. In other words, if prosodic phrasing were reliably aligned with syntactic structure, speech rate should have little effect on the proportion of prosodic boundaries since syntactic structure is maintained across rates of speech. Given this result, further research is required to understand how prosodic phrasing and speech rate interact, potentially as a function of planning scope and incrementality in language production (F. Ferreira & Swets, 2002; Levelt, 1989).

Beyond the relationship between prosodic phrasing and speech planning, the current results also highlight the relationship between prosodic and syntactic boundary strength, where prosodic boundary strength increases with the number of closing syntactic nodes, and then falls again for the highest levels of syntactic boundary strength. A similar finding by Kentner et al. (2023) was suggested to result from speech planning processes, whereby prosodic boundaries may be stronger for syntactic boundaries within larger speech units because planning effort is still required to complete the remainder of the utterance. Because syntactic boundaries within a larger utterance are typically weaker than sentence‐final boundaries, these weaker boundaries receive stronger prosodic features. Although our data do not tell us where in the utterance the syntactic boundary is located, they do indicate that prosodic phrasing is linked to the planning of speech units, which vary in syntactic boundary strength. Therefore, the cubic relationship between prosodic and syntactic boundary strengths may reflect variation in speech planning effort as the utterance unfolds, where planning effort is attenuated as speakers near the end of an utterance and begin to purge the contents of working memory. This result also challenges the idea that prosodic phrases can be classified as discrete units based on boundary strength and instead suggests that boundary strength varies across utterances as a function of planning effort and recovery processes.

In addition to the theoretical implications, these results carry for theories of prosodic phrasing, the methodology contributes to a growing body of research demonstrating the effectiveness of automatic tools for identifying prosodic and syntactic features of speech. While the current work did not independently verify the accuracy of prosodic boundary identification, the previous work by Degano et al. (2024) demonstrated that this method is able to classify strong prosodic boundaries that facilitate neural decoding of syntactic structure. This approach enables researchers to move away from idealized stimuli often used in laboratory‐based speech samples, and investigate more ecologically valid language samples. This is particularly important for developing theories of language production that can inform the creation and refinement of real‐world technologies such as interactive conversational agents and social robots. Previous research has shown that individuals prefer interactions with artificial agents that produce more human‐like speech (e.g., Kühne, Fischer, & Zhou, 2020). However, such agents are not constrained by the same cognitive and physical limitations that human speakers must routinely manage, making it important to understand how these constraints influence the prosodic structure of naturalistic speech so those features can be incorporated into communicative technologies.

The nature of the task that was used to elicit speech production in this dataset does limit our conclusions to some extent. Participants were asked to describe visual scenes out loud but not specifically to a conversational partner, thus eliminating the need to tailor their descriptions for an audience, at least at any conscious level. A remaining question concerns whether the pattern of alignment seen here between prosodic and syntactic boundaries would also be observed in interactive speech. Interestingly, our results showed poorer alignment in the situation in which speakers generated actions descriptions as opposed to simple scene descriptions. Action descriptions may involve more complex syntactic structures because they require speakers to identify relationships between direct objects and the actions that can be performed on them, which is more in line with the semantic and syntactic complexity of conversational speech. Further, Snedeker and Trueswell (2003) found that speakers tailor their use of prosodic cues for their listener when they are aware of a contextual ambiguity but often fail to use prosody to disambiguate syntactic structure when the potential ambiguity is not brought to their attention. This result suggests that, even when designing utterances for a target audience, speakers do not automatically align their prosodic phrasing to the syntactic structure but rather may have to expend deliberate effort to use prosody strategically. However, a closer comparison of prosodic phrasing across different audiences and with different communicative goals will be required to disentangle the speaker‐ and listener‐oriented aspects of the prosody–syntax interface.

A final limitation of this work concerns the identification of prosodic boundaries, which used a combination of the features pitch, duration, and energy, and therefore does not allow us to tease apart the contributions of each individual feature to overall boundary strength. We therefore cannot draw strong conclusions about whether boundary alignment changes as a function of a specific acoustic features that may contribute to prosodic boundary strength. This leaves open the possibility that only certain prosodic features can be used as cues to syntactic structure during language comprehension—for example, duration. Additionally, syntactic parsing of the speech data relied on automatic parsing carried out by the Berkeley Neural Parser. While recent technological developments allow researchers to offload labor onto automatic tools, as well as reduce researcher bias in data coding, such tools are still limited by the built‐in assumptions of the models.

Overall, these findings align with an emerging view of prosodic phrasing that suggests prosody is linked not only to features of syntactic structure but also emerges from planning and recovery processes taking place during language production (e.g., Himmelmann, 2022; Wagner & Watson, 2010). Due to the incremental nature of language production, planning requires sequential activation of relatively small conceptual units, perhaps corresponding to a single phrase. These single phrases may be the “chunks” that end up as a prosodic phrase, thus generating some degree of syntax‐prosody alignment as a byproduct of normal planning processes. Additionally, constraints such as those associated with working memory capacity may affect the number of items that can be activated at a given time. Prosodic phrasing may therefore reflect these limitations on speech planning such that each conceptual unit (which will often but not necessarily always map onto a syntactic phrase) is grouped together as a prosodic unit. Finally, these results challenge categorical approaches to prosody and suggest a need to consider not merely the presence or absence of prosodic boundaries but how continuous variation in prosodic and syntactic boundary strength reflects planning effort and influences the final form of an utterance.

## Supporting information




Supporting Information

## Data Availability

The data and analysis script corresponding to this manuscript are publicly available through the Open Science Framework and can be accessed at https://osf.io/csz89/overview.

## Notes

1 We note that whereas this speech is unrehearsed, it is not interactive. We consider this limitation in the Discussion section of the paper.

2 Each original young adult dataset consisted of 30 participants. One additional participant that was excluded from the original Action Descriptions: YA study because they could not be accurately eye‐tracked is included in the present study as our investigation does not include eye‐tracking analyses.

3 The gamma mixture model introduces randomness each time it is modeled. As a result, re‐running of the code may result in very small variations in the cutoff values separating the two types of boundaries.
