# Supplementary material for: Syntactic and Prosodic Phrasal Alignment in Naturalistic Language
Source: Cogn Sci. 2026 May 17;50:e70224. doi: 10.1111/cogs.70224 (PMC13180438; doi:10.1111/cogs.70224)
Supplement: Supplementary file 1 — Supporting Information [file COGS-50-e70224-s001.docx]

**Online Supplement**

The primary analyses reported in the paper focus on the relationship between prosodic and syntactic boundaries. Here, we further investigate the linguistic factors that predict the likelihood of prosodic boundaries in unrehearsed speech.

To begin, we identify a baseline of how well syntactic boundaries alone predict the presence of prosodic boundaries in the dataset. For each dataset, we constructed a logistic mixed effects regression model, with prosodic boundaries (1 or 0 based on the clustering from the gamma mixture model) as the dependent variable, and syntactic boundaries (contrast coded as 0.5 and -0.5 for words that did and did not represent a syntactic boundary, respectively) as the predictor variable. Participant and trial were included as random effects, except in models where trial accounted for zero variance in the model and was therefore removed (see Tables S1 and S2). A summary of the output of these models for all syntactic boundaries, as well as “strong” syntactic boundaries (i.e., words followed by at least two closing syntactic nodes beyond a word boundary) can be found in Table S1. We next calculated the predicted probability of each word being classified as a prosodic boundary. An ROC curve was calculated based on the predicted probabilities from each model, and we used Youden’s J statistic to determine a threshold for classifying words as representing prosodic boundaries based on their predicted probabilities from the model.

As can be seen in Figure S1, the logistic mixed effects regression models’ accuracy in predicting prosodic boundaries reflects a pattern that is similar to the actual alignment of prosodic and syntactic boundaries, providing additional support for our assessment that the extent of alignment between prosodic and syntactic boundaries in unscripted, unrehearsed speech is only moderate.

Table S1.

*Summary of Logistic Mixed Effects Regression Results.*

| Dataset | Measure | Estimate | SE | *Z* | *p* |
| --- | --- | --- | --- | --- | --- |
| *Any Syntactic Boundary* |  |  |  |  |  |
| Scene Descriptions: YA | Intercept | 0.04 | 0.03 | 1.29 | .197 |
|  | Syntactic boundary | 0.19 | 0.02 | 7.89 | <.001 |
| Time Pressure: YA | Intercept | 0.02 | 0.04 | 0.60 | .546 |
|  | Syntactic boundary | 0.24 | 0.02 | 10.39 | <.001 |
| Action Descriptions: YA | Intercept | -0.31 | 0.07 | -4.54 | <.001 |
|  | Syntactic boundary | 0.58 | 0.03 | 21.99 | <.001 |
| Scene Descriptions: OA* | Intercept | -0.53 | 0.02 | -22.97 | <.001 |
|  | Syntactic boundary | 0.43 | 0.02 | 17.86 | <.001 |
| *Strong Syntactic Boundaries* |  |  |  |  |  |
| Scene Descriptions: YA | Intercept | 0.33 | 0.04 | 9.27 | <.001 |
|  | Syntactic boundary | 0.70 | 0.04 | 18.64 | <.001 |
| Time Pressure: YA* | Intercept | 0.26 | 0.04 | 7.26 | <.001 |
|  | Syntactic boundary | 0.77 | 0.04 | 21.43 | <.001 |
| Action Descriptions: YA | Intercept | -0.05 | 0.07 | -0.71 | .478 |
|  | Syntactic boundary | 1.19 | 0.04 | 29.70 | <.001 |
| Scene Descriptions: OA* | Intercept | -0.29 | 0.03 | -10.45 | <.001 |
|  | Syntactic boundary | 0.86 | 0.04 | 22.40 | <.001 |

*Random effect of Trial excluded in the model.


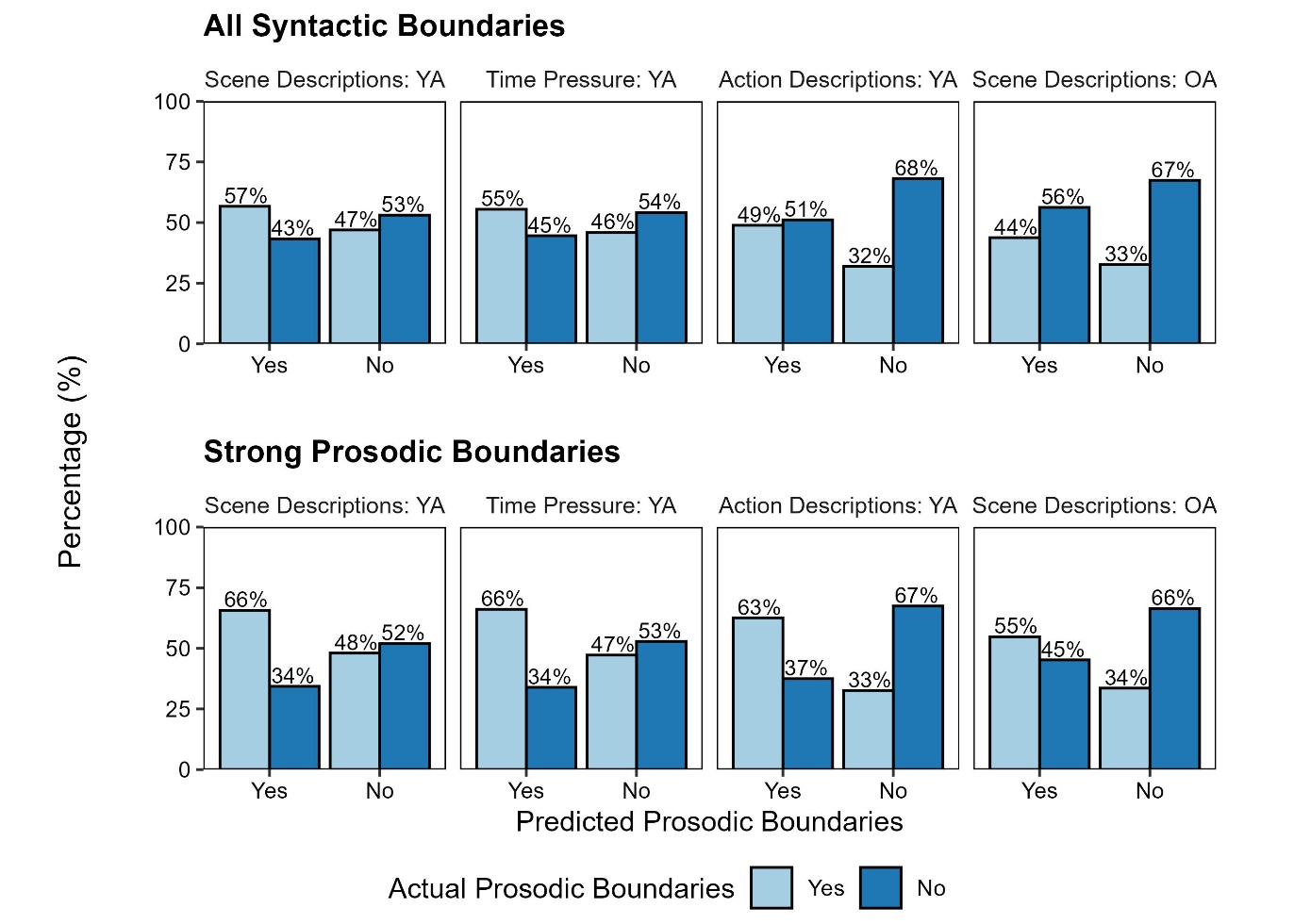


*Figure S1.* Model Accuracy in Predicting Prosodic Boundaries from Syntactic Boundaries.

Next, we consider whether the inclusion of additional variables strengthened the models’ predictions of prosodic boundaries. This analysis follows the same approach as above, but additionally includes word class (content vs. function words; contrast coded as 0.5 and -0.5 for content and function words, respectively) and position of the word in the utterance (centered and scaled). These variables were chosen because of their potential impact on the prosodic emphasis of individual words. Specifically, function words are often acoustically minimized relative to function words (Culter, 1993), and therefore may be less likely to be spoken with enough emphasis to frequently be marked as a prosodic phrase boundary. The position of each word in the utterance, as measured by the spoken order of the words, may further reflect the structure of utterances, where words produced later in each utterance are more likely to represent stronger syntactic boundaries.

Table S2 provides a summary of the model output for all syntactic boundaries, as well as words occurring at strong syntactic boundaries. Adding word class and utterance position to the models with any syntactic boundary revealed that content words were more likely to occur at the end of prosodic boundaries than function words. Interestingly, across all models, words that were produced later in the utterance were less likely to align with the edge of prosodic boundaries. This may reflect the finding that prosodic boundary strength follows a cubic relationship with syntactic boundary strength, and may suggest that words produced towards the end of an utterance, which may close off more syntactic phrases, receive less prosodic emphasis than those in the middle of utterances. Examination of the models that included strong prosodic boundaries revealed a similar trend whereby words produced later in the utterance were less likely to align with prosodic boundaries. However, in this second set of models, the effect of word class was statistically insignificant in all but one analysis (Scene Descriptions: OA). This may reflect the structure of speech, where function words overall reflect the grammatical structure of an utterance, and may be less likely to occur at the edge of a phrase (Gee & Grosjean, 1983). Finally, as seen in Figure S2, including these additional linguistic variables did not meaningfully improve the models’ predictions of which words are classified as representing prosodic boundaries.

Table S2.

*Summary of Logistic Mixed Effects Regression Results.*

| Dataset | Measure | Estimate | SE | *Z* | *p* |
| --- | --- | --- | --- | --- | --- |
| *Any Syntactic Boundary* |  |  |  |  |  |
| Scene Descriptions: YA | Intercept | -0.03 | 0.03 | -0.92 | .356 |
|  | Syntactic boundary | 0.11 | 0.02 | 4.26 | <.001 |
|  | Word Class | 0.34 | 0.03 | 12.75 | <.001 |
|  | Position in Utterance | -0.05 | 0.01 | -4.15 | <.001 |
| Time Pressure: YA | Intercept | -0.04 | 0.03 | -1.13 | .257 |
|  | Syntactic boundary | 0.16 | 0.02 | 6.71 | <.001 |
|  | Word Class | 0.29 | 0.03 | 11.08 | <.001 |
|  | Position in Utterance | -0.04 | 0.01 | -3.33 | .001 |
| Action Descriptions: YA* | Intercept | -0.36 | 0.06 | -6.20 | <.001 |
|  | Syntactic boundary | 0.53 | 0.03 | 19.90 | <.001 |
|  | Word Class | 0.27 | 0.03 | 9.97 | <.001 |
|  | Position in Utterance | -0.10 | 0.01 | -7.12 | <.001 |
| Scene Descriptions: OA* | Intercept | -0.56 | 0.02 | -25.98 | <.001 |
|  | Syntactic boundary | 0.40 | 0.03 | 15.70 | <.001 |
|  | Word Class | 0.13 | 0.03 | 4.80 | <.001 |
|  | Position in Utterance | -0.11 | 0.01 | -8.83 | <.001 |
| *Strong Syntactic Boundaries* |  |  |  |  |  |
| Scene Descriptions: YA | Intercept | 0.31 | 0.03 | 8.77 | <.001 |
|  | Syntactic boundary | 0.69 | 0.04 | 16.92 | <.001 |
|  | Word Class | 0.07 | 0.05 | 1.66 | .098 |
|  | Position in Utterance | -0.08 | 0.02 | -4.35 | <.001 |
| Time Pressure: YA* | Intercept | 0.25 | 0.04 | 7.09 | <.001 |
|  | Syntactic boundary | 0.77 | 0.04 | 19.65 | <.001 |
|  | Word Class | 0.02 | 0.04 | 0.56 | .575 |
|  | Position in Utterance | -0.04 | 0.02 | -2.32 | .020 |
| Action Descriptions: YA | Intercept | -0.07 | 0.06 | -1.44 | .253 |
|  | Syntactic boundary | 1.22 | 0.04 | 28.44 | <.001 |
|  | Word Class | -0.04 | 0.05 | -0.83 | .407 |
|  | Position in Utterance | -0.14 | 0.02 | -6.21 | <.001 |
| Scene Descriptions: OA* | Intercept | -0.27 | 0.03 | -9.57 | <.001 |
|  | Syntactic boundary | 0.91 | 0.04 | 21.71 | <.001 |
|  | Word Class | -0.10 | 0.05 | -2.25 | .024 |
|  | Position in Utterance | -0.08 | 0.02 | -4.26 | <.001 |

*Random effect of Trial excluded in the model.


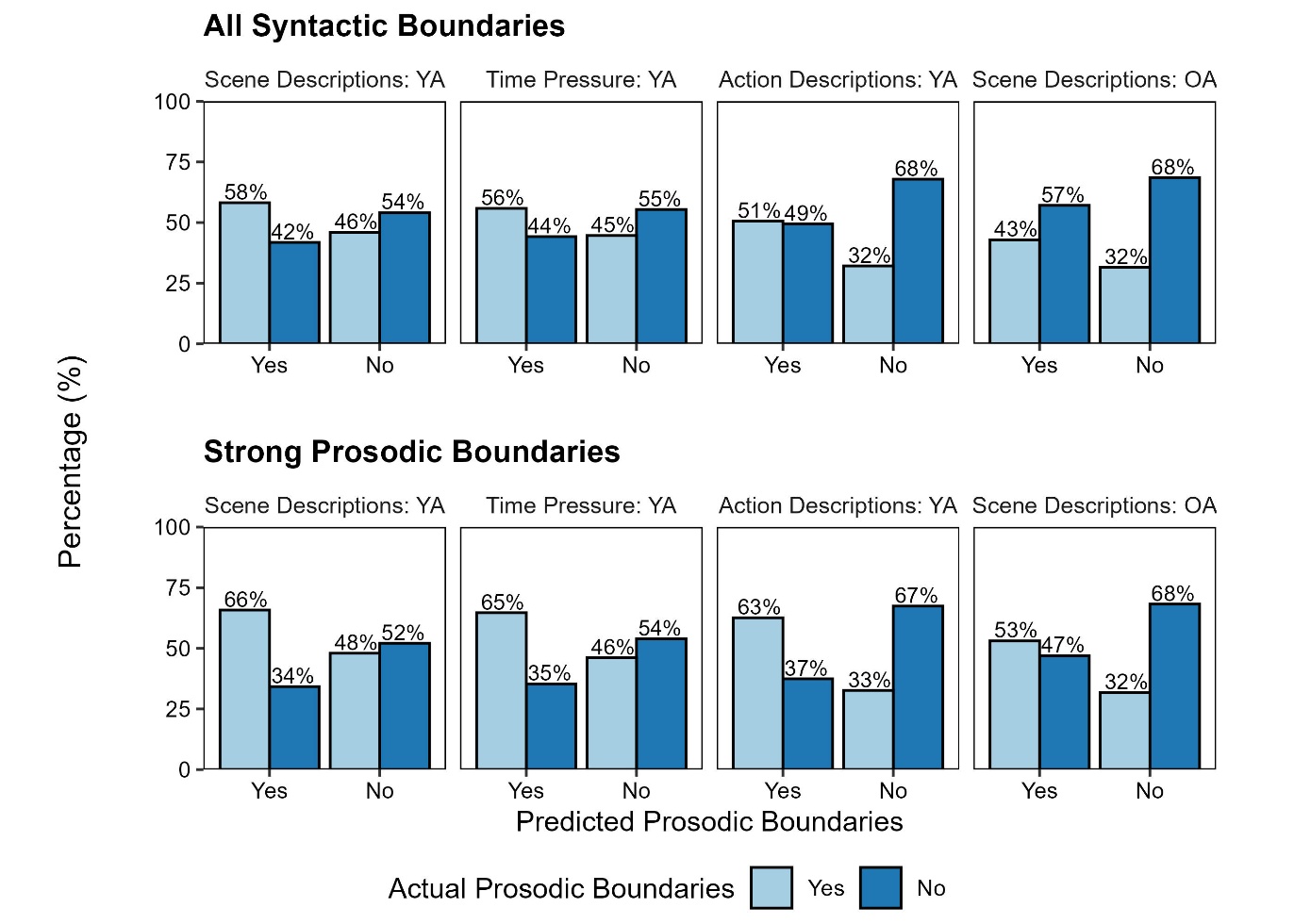


*Figure S2.* Model Accuracy in Predicting Prosodic Boundaries from Syntactic Boundaries.

Overall, the results presented here support the conclusions drawn in the main paper. Future research will be needed to determine which additional linguistic variables or cognitive constraints impact the prosody-syntax relationship. All data and analysis scripts are openly available, and researchers are free to perform additional analyses to evaluate other interesting hypotheses.
